# Folinic Acid Potentiates the Liver Regeneration Process after Selective Portal Vein Ligation in Rats

**DOI:** 10.3390/cancers14020371

**Published:** 2022-01-12

**Authors:** Jorge Gutiérrez Sáenz de Santa María, Borja Herrero de la Parte, Gaizka Gutiérrez-Sánchez, Inmaculada Ruiz Montesinos, Sira Iturrizaga Correcher, Carmen Mar Medina, Ignacio García-Alonso

**Affiliations:** 1 Department of Orthopedic Surgery, Central University Hospital of Asturias (HUCA), ES33011 Oviedo, Spain; jgutierrez066@ikasle.ehu.eus; 2 Department of Surgery and Radiology and Physical Medicine, Faculty of Medicine and Nursing, University of the Basque Country UPV/EHU, ES48940 Leioa, Spain; ignacio.galonso@ehu.eus; 3 Interventional Radiology Research Group, Biocruces Bizkaia Health Research Institute, ES48903 Barakaldo, Spain; 4 Department of Anesthesiology, Santa Creu i Sant Pau University Hospital, ES08025 Barcelona, Spain; ggutierrez024@ikasle.ehu.eus; 5 Department of Gastrointestinal Surgery, Donostia University Hospital, ES20014 Donostia, Spain; 6 Department of Clinical Analyses, Galdakao-Usansolo Hospital, ES48960 Galdakao, Spain; sira.iturrizagacorrecher@osakidetza.eus (S.I.C.); mariadelcarmen.marmedina@osakidetza.eus (C.M.M.)

**Keywords:** selective portal vein ligation, folinic acid, hepatotrophic, rodent model, future liver remnant

## Abstract

**Simple Summary:**

Fewer than 30% of patients with liver metastases are eligible for major liver resection, because liver remaining after such a surgery would be insufficient to cover the patient’s needs; this is called a low percentage of future liver remnant (FLR). Folinic acid (FA) has been shown to play a crucial role in cellular synthesis, regeneration, and nucleotide and amino acid biosynthesis. The aim of this piece of research was to evaluate the effect of FA as a potential hypertrophic hepatic enhancer agent after selective portal vein ligation (PVL) to ensure adequate FLR. We have confirmed in our rodent model that FA accelerates liver regeneration after PVL and enhances recovery of liver function. These findings may allow more patients to be eligible for liver resection without jeopardizing postoperative liver function.

**Abstract:**

Liver resection remains the gold standard for hepatic metastases. The future liver remnant (FLR) and its functional status are two key points to consider before performing major liver resections, since patients with less than 25% FLR or a Child–Pugh B or C grade are not eligible for this procedure. Folinic acid (FA) is an essential agent in cell replication processes. Herein, we analyze the effect of FA as an enhancer of liver regeneration after selective portal vein ligation (PVL). Sixty-four male WAG/RijHsd rats were randomly distributed into eight groups: a control group and seven subjected to 50% PVL, by ligation of left portal branch. The treated animals received FA (2.5 m/kg), while the rest were given saline. After 36 h, 3 days or 7 days, liver tissue and blood samples were obtained. FA slightly but significantly increased FLR percentage (FLR%) on the 7th day (91.88 ± 0.61%) compared to control or saline-treated groups (86.72 ± 2.5 vs. 87 ± 3.33%; *p* < 0.01). The hepatocyte nuclear area was also increased both at 36 h and 7days with FA (61.55 ± 16.09 µm^2^, and 49.91 ± 15.38 µm^2^; *p* < 0.001). Finally, FA also improved liver function. In conclusion, FA has boosted liver regeneration assessed by FLR%, nuclear area size and restoration of liver function after PVL.

## 1. Introduction

Despite advances in chemotherapeutic approaches and other techniques for the ablative treatment of liver tumors, liver resection remains the gold standard treatment for colorectal cancer liver metastases (CRCLM) or other liver malignancies [1,2,3,4]; CRCLM are the main indication for liver surgery in western countries [4,5]. Most of them have a gastrointestinal origin, which can be explained since the tumor cells can migrate from the primary tumor through the blood vessels to the liver, establishing and developing new tumor implants in this organ [4]. This also explains why a high percentage of patients with primary tumors of the gastrointestinal tract also present CRCLM at the time of diagnosis (synchronous metastases) [2,3,4].

Different studies indicate that up to 75% of the hepatic parenchyma could be safely removed without compromising liver function in patients with a good hepatic functional status, increasing the risk of liver failure when the liver remnant is less than 25%. Whereas in patients with a jeopardized hepatic function, the future liver remnant (FLR) should not be less than 40% [2,4,6,7]. After a partial hepatectomy in which the FLR is insufficient, so-called “small for size” syndrome (SFSS) will occur. SFSS is defined by the presence of prolonged cholestasis, coagulopathy, and ascites in the absence of ischemia [8]. Therefore, it is important to ensure a sufficient FLR or to increase it, as a previous stage to liver resection, in order to meet the patient’s metabolic requirements, and this is achieved with techniques that induce hepatic hypertrophy.

In recent years, multiple surgical strategies have been developed to ensure sufficient FLR. Portal embolization (PE) or selective portal vein ligation (PVL), for example, are intended to selectively occlude the portal lumen of one or several hepatic segments, triggering hepatocyte proliferation to achieve compensatory hypertrophy of the rest of the organ, which reduces the risk of liver failure after liver resection [2,4,9]. Usually, this compensatory hypertrophy requires a few weeks, and less than 5% hypertrophy is a prognostic indicator of post-operative hepatic insufficiency [2]. While PE can be performed using interventional radiology techniques, PVL is performed through a surgical intervention first, and then the liver resection is performed in a second surgery [2,3,10]. Damage caused by the first surgery, such as anatomical destructuring or fibrosis, may complicate the second procedure, increasing the morbidity and complication rate compared to PE [2,10].

In 2012, Schnitzbauer et al. reported the first cases of a novel interpretation of two-staged resection [11], the so-called associating liver partition and portal vein ligation for staged hepatectomy (ALPPS). ALPPS associates PVL with the partitioning of the ligated hepatic segment. Thus, this process achieves a faster hepatic hypertrophy than other surgical techniques, up to an 80% increase in 7 days [2,3,6,10]. However, the ALPPS technique has also been associated with higher postoperative morbidity and mortality rates; even though these complications rates may be mitigated with the improvement of the surgical experience and a better selection of the patients to be treated [12].

Beyond these surgical techniques to increase the FLR, non-surgical options to obtain a greater and faster growth of the FLR have been evaluated at an experimental level in the last few years. An example of these approaches are the studies based on the administration of mesenchymal stem/progenitor cells (MSC) [13,14,15], induction of hypoxia [16,17,18], or even pharmacological enhancement of hepatocyte regenerative response, such as folinic acid (FA) [19,20]. After partial hepatectomy, FA was shown to increase liver regeneration [20]. Therefore, based on this previous experience, our current research proposes to evaluate the effect of FA as a hypertrophic hepatic enhancer agent after selective PVL in a rat model.

## 2. Materials and Methods

This project was approved by the Animal Experimentation Review Board (CEEA) (ref.: M20/2018/023), as well as the Review Board on Research with Biological Agents (CEIAB) (ref.: M30/2018/024) of the University of the Basque Country (UPV/EHU). It was conducted in accordance with national and European regulations on animal experimentation and in accordance with the ARRIVE guide.

A total of 64 male WAG/RijHsd rats, aged 3 months and weighing approximately 250–280 g, were used. The animals were housed with 12 h circadian light/dark cycles and *ad libitum* food and water intake. The animals were uniformly distributed in eight different groups: seven experimental groups and one control group, with eight animals assigned to each group. Table 1 shows a summary of the procedures carried out on each of the different experimental groups.

### 2.1. Selective Portal Vein Ligation

Under 1.5% isoflurane anesthesia, the animal was placed in the supine decubitus position, the abdomen was shaved and the surgical site was aseptized with 70% alcohol. A median laparotomy was performed and, aided by four retractors, the left lateral lobe (LLL), the right and the left portion of the median lobe (RML and LML), and some small intestinal sections were pulled out from the abdominal cavity, and then covered with 37 °C saline-moistened gauze to reduce their desiccation, in order to minimize the risk of future adhesions.

**Figure 1:**
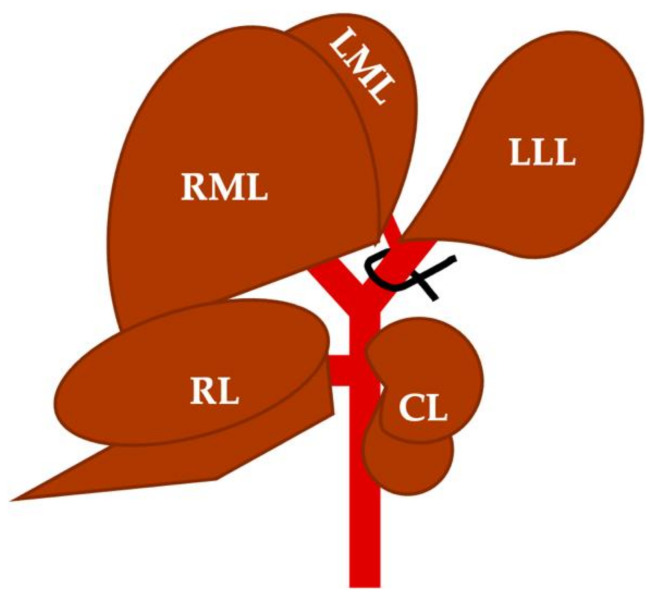
Schematic view of the visceral surface of the rat liver with details of the lobar segmentation of the hepatic parenchyma (right portion of the medial lobe [RML], left portion of the medial lobe [LML], right lobe [RL], caudated lobe [CL], and left lateral lobe [LLL]), and the distribution of the portal venous system. It also shows the ligature placement site, demonstrating how it compromises the portal irrigation of the LLL and LML.

By placing a ligature on the left branch of the portal vein, portal venous flow to LLL and LML was interrupted (the sum of the liver mass of both lobes represents approximately 45–50% of the total liver mass) (Figure 1).

**Figure 2:**
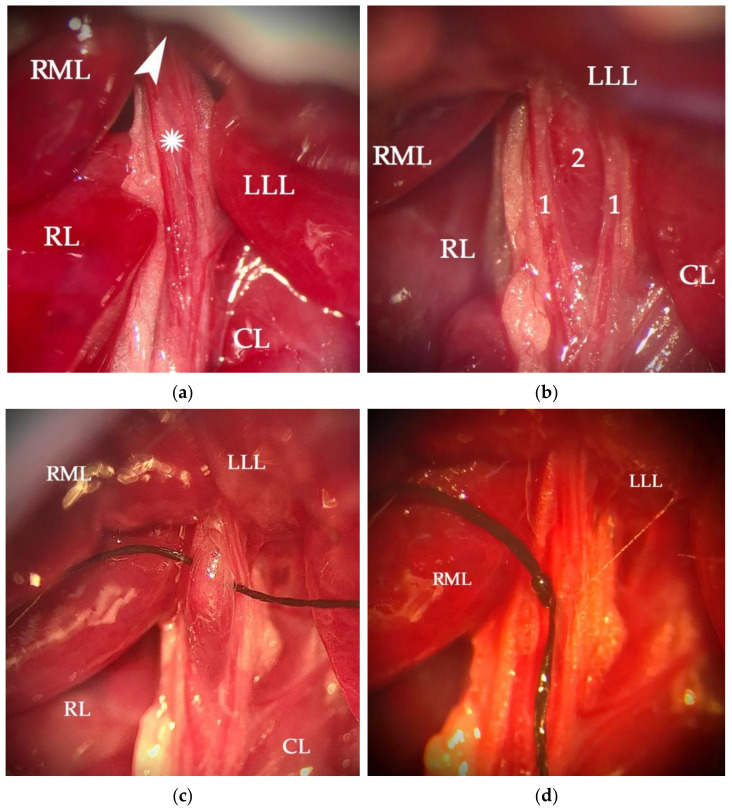
Microscope photographs of the hepatic hilium at its entrance to the left lateral lobe (LLL). Visualization at 10× magnification of the hepatic pedicle (asterisk) surrounded by the different lobes: right portion of the medial lobe (RML), right lobe (RL), caudated lobe (CL), and LLL (**a**). The arrowhead marks the entry zone of the left portal branch into the LLL. Detail of the left portal branch (2) and the dissected arteries and bile ducts (1) on both sides (**b**). 6/0 silk ligature slipped behind the left portal vein branch, avoiding arteries and bile duct (**c**). Portal vein branch of the LLL occluded with ligature (**d**).

On the posterior side of the liver, the portal hilum was located (Figure 2a) and the portal vein was identified. Under a surgical microscope, the hepatic arteries and biliary ducts were carefully isolated, dissecting the left portal vein at its entry into the LLL (Figure 2b). Then, using a blunt micro Deschamps, a 6/0 silk strand was guided under the left portal vein (Figure 2c). Prior to tying the ligature, it was ensured that no arterial or biliary branches were compromised; then, the ligature was knotted (Figure 2d).

Once the absence of bleeding from any structure was verified, the small intestine, the LLL and the RML, and the LML were re-introduced into the abdominal cavity. Right after, 1 to 1.5 mL of saline at 37 °C was instilled into the abdominal cavity and the incision was closed by layers; the muscular layer was closed with 4/0 polypropylene, and the skin was closed with 3/0 silk.

### 2.2. Folinic Acid or Saline Administration

Once the PVL was accomplished and the surgical incision was closed, saline or FA was administered via femoral vein.

A small incision in the inguinal region was performed in order to expose and identify the femoral vein. Then, 1 mL of saline or FA solution (2.5 mg/kg) was slowly administered through a 30 G needle. When fluid infusion was accomplished, the needle was removed and hemostasia was applied (1–2 min); then, the skin was closed with single 3/0 silk stiches.

Finally, just before removing the anesthesia, a subcutaneous injection of meloxicam (5 mg/mL) was given to ensure postoperative analgesia. Post-surgical recovery was completed in a clean cage with a heating system at 22 °C.

### 2.3. Blood Collection and Histological Samples Obtention

At the end of the different time periods established for each experimental group, blood samples were collected, and liver tissue was extracted to measure the weight and volume and to obtain tissue for histological study.

Under isoflurane 1.5% anesthesia, the abdominal cavity was opened, and the inferior vena cava was exposed and punctured with a 20 G needle to extract as much blood as possible (5–6 mL). To obtain the serum, the blood was introduced into SST™ II Advance tubes (BD Vacutainer^®^, BD, Franklin Lakes, NJ, USA. Ref.:367955) and centrifuged for 10 min at 3000 rpm. The extracted serum was frozen until analysis.

Regarding liver tissue, the entire liver was removed. Then, the weight was also recorded, both the total and the atrophic and hypertrophic lobes separately. FLR percentage (FLR%) was calculated by dividing the FRL (in mg) by the total functional liver weight (TLW, in mg). It was expressed as a percentage (FLR% = FLR × 100/TLW). Once all measurements were taken, the liver tissue was fixed in 4% paraformaldehyde solution.

### 2.4. Serum Samples and Histological Sections Analysis

Serum samples were analyzed in a Cobas^®^ 8000 module c702 analyzer for clinical use and the following parameters were determined, all using commercial kits for clinical use: alanine aminotransferase (ALT) (Ref. 05880797 190), aspartate aminotransferase (AST) (Ref. 05880819 190), alkaline phosphatase (ALP) (Ref. 05166888 190), total bilirubin (TBil) (Ref. 05795419 190), and albumin (Ref. 0005166861 190), all from Roche (Roche Diagnostics GMBH, Mannheim, Germany).

Microscopic assessment of hypertrophied liver tissue was performed on transverse sections of the RML. The samples were embedded in paraffin and 5 µm histological sections were stained with hematoxylin/eosin. The stained sections were studied under light microscopy and photographs of five random fields were obtained at 40× to measure at least 100 hepatocytes per animal. The area quantification was performed, in a randomized and blinded manner, by an independent researcher. For this purpose, once the hepatocyte nuclei were identified, it was surrounded with the proper tool for area measurement of Leica Application Suite (LAS) (Leica Application Suite software, Leica Microsystems, Wetzlar, Hesse, Germany); then the mean and the standard deviation were calculated.

### 2.5. Statistical Analysis

Once normality of the data sets was verified, all the parameters obtained were represented by the mean and standard deviation (SD). The comparison between the different experimental groups was carried out using analysis of variance tests (ANOVA), accepting a statistical significance level of 95% (*p* < 0.05). If statistically significant differences were found between the experimental groups, multiple comparison tests were also performed (Tukey’s multiple comparison test for between-groups comparison). All analyses were performed with GraphPad Prism 8.2.1 (GraphPad Software, San Diego, CA, USA).

## 3. Results

In each and every animal subjected to PVL, selective ligation of the left portal vein induced some degree of LLL atrophy, with different levels of compensatory liver hypertrophy according to the experimental groups. Bleeding due to small lacerations in the hepatic parenchyma during the dissection of the periportal tissues or due to ruptures of arterioles or capillaries was the main complication during the process. These bleedings were easily managed by direct hemostasis with cotton swabs. No postoperative complications were observed.

### 3.1. Regenerative Response of the Liver after Selective PVL

**Figure 3:**
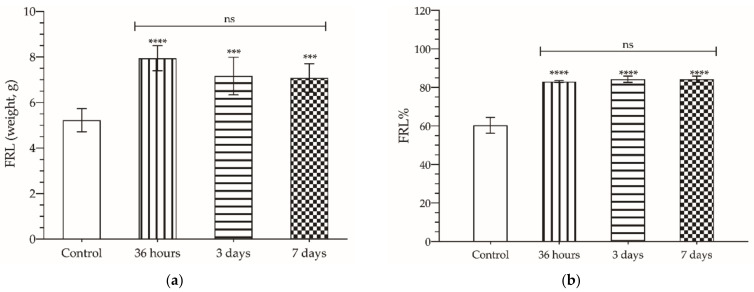
Mean weight (**a**) of future liver remnant (FLR) parenchyma and percentage of FLR mass relative to total liver mass (FLR%) (**b**) 36 h, 3, and 7 days after selective portal vein ligation (PVL); control refers to healthy rats, not subjected to any surgical intervention or treated with any substance. The asterisks show statistically significant differences compared to the control (***: *p* < 0.001; ****: *p* < 0.0001). The upper bar shows the significant differences between the groups indicated by it (ns: *p* > 0.05).

Following PVL, compensatory FLR hypertrophy was observed, peaking at 36 h (7.95 ± 0.55 g) when up to 40% increment in FLR mass was registered. From then on, no significant changes were found (3rd day: 7.17 ± 0.83 g; 7th day: 7.08 ± 0.62 g; *p* > 0.05) (Figure 3a,b).

If we focus on the whole liver, there was no increase in weight, either at 36 h, or at 3 or 7 days (9.82 ± 0.82, 7.97 ± 0.66, and 8.15 ± 0.54, respectively) compared to the control group (8.69 ± 0.99 g; *p* > 0.05). Since no significant differences were found when comparing the 36-h, 3-day and 7-day groups, we decided, in accordance with the 3R principle (reduction, refinement, and replacement), not to test the hepatotrophic effect of FA on the 3rd day, and to study its effect only in the 36-h and 7-day groups

### 3.2. Enhancing Effect of FA on Hepatic Hypertrophy after Selective PVL

**Figure 4:**
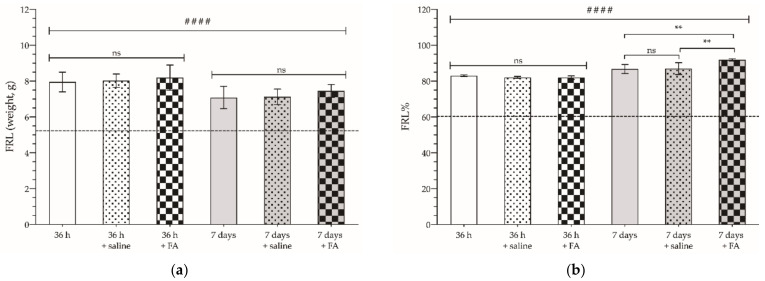
Mean weight (**a**) of future liver remnant (FLR) parenchyma and percentage of FLR mass relative to total liver mass (FLR%) (**b**) 36 h (white bars) and 7 days (gray bars) after selective portal vein ligation (PVL), of untreated (no pattern bars), saline-treated (dotted pattern), or 2.5 mg/kg folinic acid (FA)-treated (checkered pattern) animals. The dotted line indicates the mean weight of the FLR equivalent before PVL. The pads show the statistically significant differences compared to the basal weight of FLR (####: *p* < 0.001), and the asterisks show the statistically significant differences between the groups indicated by the upper bar (**: *p* < 0.01); ns—*p* > 0.05.

First, it was verified that saline administration (the medium in which the FA treatment was administered) did not induce changes in hepatic hypertrophy 36 h or 7 days after selective PVL (*p* > 0.05), either in the weight of the FLR (Figure 4a), or in the percentage of FLR relative to the total weight of the liver (Figure 4b). Following FA administration, no effect on the amount of regenerated liver mass was observed after PVL either at 36 h, or on the 7th day. However, analyzing the FLR% after 7 days, we could observe a slight but significant increase in the percentage of regenerated liver mass (91.88 ± 0.61%) compared to the control group or the saline-treated group (86.72 ± 2.5 vs. 87 ± 3.33%; *p* < 0.01).

**Figure 5:**
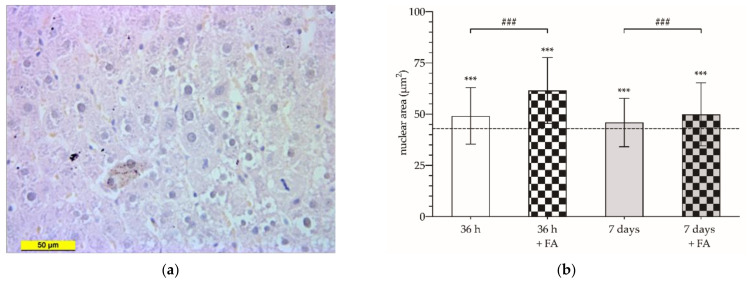
Representative histological section stained with Hematoxylin-Eosin of the hypertrophied liver parenchyma; the scale bar corresponds to 50 µm (**a**); and mean values of the hepatocyte nuclear area in squared micrometers (µm^2^) of animals subjected to selective portal vein ligation (PVL) and analyzed after 36 h (white) or 7 days after PVL (gray), treated or not with folinic acid (FA) (square or smooth pattern, respectively); the dotted line represents the mean value of the nuclear area of the control group (**b**). The asterisks show the statistically significant differences compared to the control group (***: *p* < 0.001), and the pads show the statistically significant differences between the groups indicated by the upper bar (###: *p* < 0.001).

Under microscopic examination of liver histological sections (Figure 5a), we could also confirm the pro-hepatotrophic effect of FA by measuring the nuclear area of hepatocytes. The hepatocyte nuclear area of animals not subjected to selective PVL was 41.69 ± 14.05 µm^2^. These figures were increased by approximately 20% and 10% after 36 h and 7 days (49.18 ± 13.79 and 45.95 ± 11.83 µm^2^, respectively; *p* < 0.001). 36 h after PVL, FA administration induced an almost 45% increase in hepatocyte nuclear area compared to control figures (61.55 ± 16.09 µm^2^; *p* < 0.001) and a 25% increase compared to animals subjected only to PVL (49.18 ± 13.79 µm^2^; *p* < 0.001). The nuclear area quantified after 7 days was also higher in FA-treated animals; however, this increase did not exceed 20% compared to the control (49.91 ± 15.38 µm^2^; *p* < 0.001) (Figure 5b).

**Figure 6:**
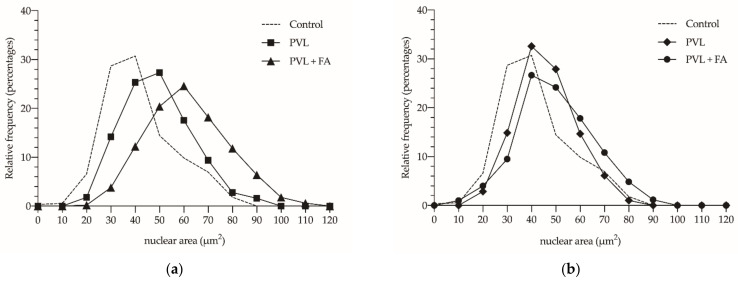
Histogram of relative frequencies of nuclear area quantified in control animals (dashed line) and in animals subjected to selective portal vein ligation (PVL) (solid line) and analyzed 36 h after PVL (**a**) (square—untreated animals; triangle—animals treated with folinic acid (FA)); or analyzed after 7 days of evolution (**b**) (rhombus—untreated animals; circle—animals treated with FA).

Figure 6 shows the relative frequency histogram of the nuclear area measured. It can be shown that, 36 h after PVL (Figure 6a), there was a frequency shift, demonstrating a major number of larger nuclei, a fact that is even more accentuated with the FA treatment. After 7 days (Figure 6b), we could see how the relative frequencies of the control group and the PVL and PVL + FA groups overlap, reinforcing the previous results, without noticing any differences between the experimental groups.

### 3.3. Liver Functional Status after Selective PVL

**Figure 7:**
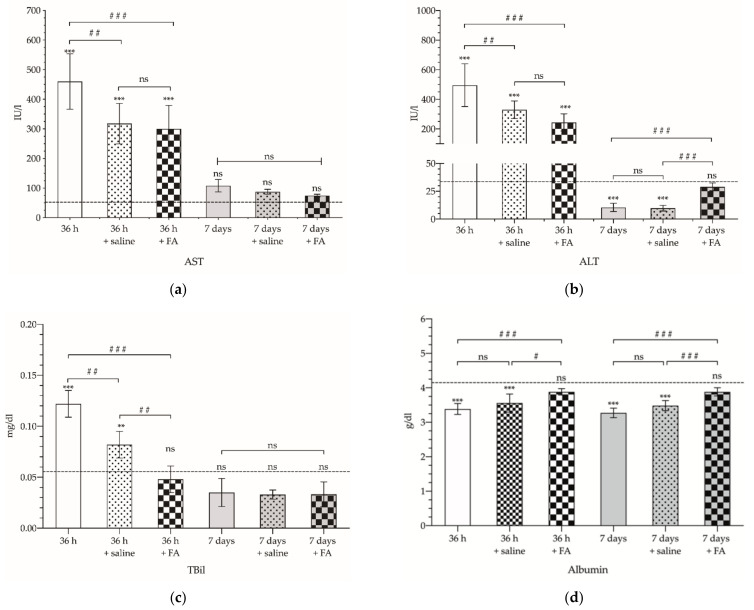
Aspartate aminotransferase (AST) (**a**), alanine aminotransferase (ALT) (**b**), total bilirubin (TBil) (**c**) and albumin (**d**) serum levels detected in serum samples from animals subjected to selective portal vein ligation (PVL) and without treatment (no pattern), intravenous administration of saline (dotted pattern), or with intravenous administration of 2.5 mg/kg folinic acid (FA) (checkered pattern), and analyzed 36 h (white) or 7 days (gray) after PVL. Units are expressed in international units per liter (IU/L) for AST and ALT, milligrams per deciliter (mg/dL) for TBil, and grams per deciliter (g/dL) for albumin. The asterisks show the statistically significant differences compared to the control group (**: *p* < 0.01; ***: *p* < 0.001), and the pads show the statistically significant differences between the groups indicated by the upper bar (#: *p* < 0.05; ##: *p* < 0.01; ###: *p* < 0.001); ns: *p* > 0.05.

At the same time, biochemical analysis was performed to determine the serum levels of several biomarkers in serum samples. Firstly, a marked increase in AST (Figure 7a) and ALT (Figure 7b) was observed as early as 36 h after PVL (460 ± 93.63 and 496.2 ± 144.4 IU/L, respectively). For both enzymes, it was also observed that, either in those animals that received saline serum or in those treated with FA, the increase in serum levels was lower, with no significant differences for the FA treatment (*p* > 0.05). For AST, determinations performed 7 days after PVL show that the enzyme values decrease, almost reaching the basal values (*p* > 0.05). In contrast, AST levels detected after 7 days presented a marked decrease, dropping below the reference values (10.50 ± 3.73 IU/L; *p* < 0.001); saline infusion did not significantly modify these figures. However, FA treatment was able to reverse this reduction (29 ± 3.52 IU/L; *p* > 0.05).

TBil also showed a marked increase 36 h after PVL (Figure 7c). The administration of saline reduced the TBil values by 40% (0.082 ± 0.013 mg/dL); however, FA was able to decrease serum TBil to values similar to those detected in the control group (0.048 ± 0.013 vs. 0.053 ± 0.010 mg/dL, respectively; *p* > 0.05). On the 7th day after PVL, TBil values were within normal range in all of the groups, and no significant differences were observed as a consequence of saline or FA administration (0.033 ± 0.005 vs. 0.033 ± 0.012 mg/dL; *p* > 0.05).

Finally, in contrast to those observed for the other parameters analyzed, albumin showed lower serum levels 36 h and 7 days after selective PVL (3.3 ± 0.14 and 3.4 ± 0.16 g/dL, respectively; *p* < 0.001). Saline-treated animals did not show significant differences compared to non-treated animals (*p* > 0.05), whereas FA administration led to a normalization of albumin values both at 36 h and 7 days (3.9 ± 0.093 and 3.9 ± 0.12, respectively) (Figure 7d).

## 4. Discussion

Major liver resection, which commonly involves the resection of four or more liver segments [21], is the gold standard treatment for primary and secondary liver malignancies, but commonly less than 30% of patients are eligible for this approach because of different comorbidities or a high number or size of tumor implants. For example, in those patients with mild steatosis, cholestasis and early cirrhosis (Child–Pugh A), or severe steatosis and cholestasis, major hepatectomy is only recommended when the FLR is above 30–35% or 40%, respectively; in patients with normal liver function, an FLR of at least 20% can be considered [22]. However, in those patients graded as Child–Pugh B or C plus portal hypertension, even small liver resections can result in severe post-hepatectomy hepatic insufficiency or failure [23].

Though PVL is currently applied in cases of inadequate liver remnant volume to induce hypertrophy, it cannot be used in all kinds of patients. Many groups reject the indication for patients with impaired hepatic regenerative capacity, such as cirrhotic patients or chronic alcohol consumption [24,25,26]. But also, there are concerns because of the mortality rates associated with these techniques, as well as some uncertainty about the influence of PVL on oncologic response and outcome [2,3,6,7,10,27]. To overcome the undesired effect of surgery on tumor progression, some authors propose adding chemotherapy to PVL. Fisher et al. reviewed the outcome of patients with colorectal cancer liver metastases treated with selective portal vein embolization. They compared those patients who underwent chemotherapy between embolization and hepatic resection versus those who did not receive chemotherapy, finding a lower rate of disease progression in those who received chemotherapy [28].

Thus, experimental studies are still needed to find strategies that enhance liver regeneration without compromising the functional status of the liver or enhancing the growth of tumor implants. For this purpose, some key points are the choice of the experimental model and the study time after the tested surgical procedure. Most of these studies have been carried out in a rat experimental model [29,30,31,32,33], although others have opted for rabbits [34], or even pigs [35]. In most of the studies, the study period ranges from 24 h to 7 or 8 days after the surgical procedures, which is the same period used in our model. Even though some studies refer to 3 days [31,36,37] as the point of peak hypertrophy, in our model we have observed that hypertrophy reached its maximum as early as 36 h after selective PVL. This is consistent with the studies of Higgins and Anderson, who had demonstrated that, after partial hepatectomy, the greatest increase in liver mass occurred in the first 24–48 h [38].

In this context, several approaches have been proposed to improve the liver regeneration process before partial hepatectomy is performed; some of them are already being used today in hepatic surgery for tumor resection, such as ALPPS. Liao et al. carried out these studies in an ALPPS model in rabbits, arguing that the anatomical differences between the rat liver and human beings might make it an inappropriate model and the results may not be applicable to humans. They compared the results of ALPPS with selective PVL, and demonstrated that, at 7th day, ALPPS achieved a significantly higher percentage of FLR than PVL (27.5 ± 7.3% vs. 20.3 ± 7.3%, respectively) [34]. Unfortunately, these favorable results in terms of FLR did not correlate with the serum levels of the liver markers analyzed after 3 days, since the different parameters showed worse values in those animals subjected to ALPPS compared to those subjected only to selective PVL.

García-Pérez et al. obtained similar results in rats when comparing selective PVL with ALPPS, demonstrating that the association of PVL with in situ liver partitioning induced a significantly greater increase in both liver volume and weight as early as 48 h, the effect being maintained up to 12 weeks [33]. Their figures are also higher than those reported in this piece of work. In contrast, selective PVL was shown to have less effect on altering serum AST, ALT, and TBil values. Within 24 to 48 h after ALPPS, AST and ALT reached significantly higher values than those found after selective PVL (values higher than 6000 and 3000 IU/L, respectively), which went back to normal progressively until the 8th day. TBil was also elevated (values higher than 0.2 IU/L), and these levels were maintained after eight days (*p* > 0.05, compared to control animals). A similar behavior was observed in our PVL model; liver transaminases (AST and ALT) remained elevated after 36 h without reaching normal levels after 7 days.

Another remarkable fact observed in our data set is that those experimental groups that only received saline infusion and were analyzed 36 h after PVL, showed a significant amelioration in AST, ALT, and TBil values. It is important to point out that blood loss occurs during surgery, even if only a small amount, which can lead to acute hypovolemic shock, with the subsequent decrease in intravascular volume resulting in organ damage, such as the liver [39]. It is known that fluid therapy, either with colloidal solutions, plasma, or crystalloid fluids such as saline, allows a prompt recovery of liver function, as well as the reduction of comorbidities after major liver surgeries. Therefore, the restitution of volemia with the addition of 1 mL of saline after PVL could explain why the figures of the serum markers analyzed are better than the ones obtained after PVL without saline administration [40].

Dili et al. also analyzed the differences in liver hypertrophy induced by ALPPS or selective PVL after 2, 3, and 7 days. As with the previously mentioned authors, they also demonstrated that the ALPPS technique achieved a higher FLR on the 2nd and 3rd day, but not on the 7th day [37]. They also reported a significant release of AST and ALT on day 1 after ALPPS and PVL, with no differences between the two techniques; however, they did not provide further data regarding the subsequent days. Finally, the data published by Sheng et al. and Shi et al. were in line with the previous studies [32,41]. Therefore, this previously reported evidence indicates that there is no direct relationship between the consecution of a good volume of FLR and the adequate functional status of the resulting hepatic tissue, preventing liver failure and the increased mortality and morbidity that has been seen in studies of this technique [42,43,44].

Other experimental approaches [13,14,15,16,17,18,19] associating other treatments to PVL with hepatectomy, such as the use of mesenchymal stem cells (MSC), have shown promising results. Khuu et al. injected Adult Human Liver Mesenchymal Stem/Progenitor Cells (ADHLSCs) into the spleen of SCID mice and studied the migration of cells from the splenic site of injection to the liver parenchyma [13]. Their results showed that, as early as 10 min after cell transplantation, isolated viable individual cells or clusters were evident mainly in the periportal structures of the recipient liver. Furthermore, they were able to detect viable and differentiated cells in the livers of recipient mice up to 60 days after transplantation. Microscopic evaluation of immunostained liver sections revealed a morphology similar to that of hepatocytes, showing that transplanted nondifferentiated ADHLSCs are able to contribute to mouse liver regeneration after partial hepatectomy. Similar results were published by Wabitsch et al. [14]. After 70% partial hepatectomy, those mice receiving MSC showed a significantly higher hepatocyte and sinusoidal endothelial cell proliferation. Other than this, mice receiving MSC showed a significantly lower loss of body weight. Transaminases levels were also lower in MSC-treated animals 3 and 5 days post-operation. Serum albumin levels did not show significant changes, and serum TBil was below detection limits. In a rat model of partial hepatectomy, Li et al. also achieved similar results [15]. Intra portal injection of bone marrow-derived MSC, performed 24 h after 70% partial hepatectomy, enhance liver regeneration.

Schadde et al. suggested that hemodynamic changes after PVL or ALPPS and the resulting hypoxia may explain liver regeneration; they found that exacerbation of hypoxia in the PVL group promoted liver regeneration, whereas enhancement of hypoxia in the ALPPS group suppressed regeneration. They also demonstrated that pharmacologically induced hypoxic signaling (by dimethyloxaloglycine (DMOG) administration, a prolyl-hydroxylase inhibitor) accelerates liver regeneration [16]. These results are in accordance to previously published studies by Maeno et al. and Schmeding et al. [17,18], that described a notable release of hypoxia-inducible factor 1α (HIF-1α) in regenerating liver after partial hepatectomy. Ohtake et al. also analyzed effect of drug administration, omeprazole, on hepatocytes proliferation. They observed that, after omeprazole administration, liver regeneration was enhanced, and they hypothesized that this phenomenon could be mediated by an increase of the gastrin levels [19].

In addition to registering liver weight, Ki-67 labeling is commonly used as an indicator of cell proliferation in liver parenchyma not subjected to PVL, as well as the counting of the number of nuclei in mitosis. In those studies that analyzed the differences in liver regeneration between selective PVL and ALPPS, a significantly higher number of hepatocytes positively labeled for Ki-67 was detected in the latter cases [15,29,31,32,33,34,35,36,37]. In our study, because of the impossibility of conducting Ki-67 immunohistochemical studies, we used the measurement of the nuclear area of hepatocytes as an indirect measure of their proliferative/regenerating state. This assessment is supported by the studies of Wang et al. and Fausto et al., who demonstrated that the increase in nuclear area is directly related to the increase in the amount of genetic material [45,46]. In addition, it also allows for a simple determination of the proportion of polyploid nuclei.

In our study, as in others already published, we observed a significant increase in FLR% after selective PVL. Whereas FA-treated animals had an increase in FLR% of 54.96 ± 9.96% compared to animals only subjected to PVL, those receiving saline had a lower increase in FLR% of 45.26 ± 9.66 (*p* < 0.01). It is interesting to note that this positive effect of FA was only observed when analyzing FRL%, not when weight per se was considered. The reason for this phenomenon lies in the fact that, once the liver reaches its maximum mass of around 7–8 g, no further increase in size can be observed. However, when considering the hypertrophied liver mass after PVL in relation to the total liver mass (hypertrophied + atrophic liver mass), differences can be observed.

Our therapeutic approach with FA allows us to associate the increase in FLR with slightly lower changes in the hepatic serum markers analyzed both at 36 h and at 7 days. It is true that most of the previous studies have shown that liver enzyme levels return into normal levels 7 to 14 days later without any specific treatment [30,31,32,33,36]. However, our experimental treatment with FA achieves significantly better values as early as 36 h in most of the analyzed parameters. We hypothesize that these better outcomes obtained as a result of FA administration are based on the crucial role that this compound plays in proper cellular function, especially related to cellular synthesis, repair and regeneration (DNA synthesis, repair, and methylation, as well as nucleotide and amino acid biosynthesis), as well as energy production (ATP) and protein synthesis, and the production of immunomodulatory molecules, inosine, and adenosine [47,48,49].

This phenomenon was already observed years ago in a study published by our research group. Portugal et al. compared the efficacy of cyclosporine, superoxide dismutase (SOD), allopurinol and FA in enhancing hepatocyte regeneration after hepatic ischemia in rats [50]. First of all, Portugal found that the peak in liver regeneration was at 24 h, which was quite close to that determined in this study (36 h). Furthermore, he showed that FA (also cyclosporine and SOD) significantly increased the intensity of the regenerative response in the liver. This increase was defined in terms of mitotic hepatocyte number, but not in an accelerated rate of hepatic regeneration.

Our experimental approach, which involves the use of FA to accelerate liver regeneration, could have another potential therapeutic effect at the same time. It has long been known that FA increases the cytotoxicity of chemotherapeutic agents against certain tumor lines of colorectal cancer, such as fluorouracil [51]. Therefore, in our opinion, it is interesting to study these aspects in more detail, and once its potential as a hepatic hypertrophy-inducing agent has been proven, it should also be studied together with its antitumor effect.

A limitation of our study is that we only tested a single dose of FA and at a single time. Some studies indicate that a diet rich in FA (40 mg FA/kg diet) for 4 weeks tends to increase hepatocyte division and improve liver morphology in aged rats [52]; however, it is important to take into account that other authors, such as Marsillach et al. [53], have shown that moderately high doses of FA (25 mg/kg) exacerbate liver fibrosis in rats. In addition, it would also be interesting to analyze whether this effect of the FA is maintained in those cases of major PVL (70–90%).

## 5. Conclusions

FA administration improves serum values of liver function markers as early as 36 h after PVL and, in some of them (TBil and albumin), leads to their normalization. In addition, it also allows that, both at 36 h and on the 7th day, the nuclear area of the hepatocytes was larger, indicating higher DNA replication. However, not until the 7th day is the effect on the increase of FLR% evident.

These data suggest that FA could be used as a liver regeneration booster, in association with PVL, as a previous surgical procedure to CRCLM resection in patients with an insufficient FLR.

## Figures and Tables

**Table 1:** Summary of experimental groups.

Group No.	Group Name	PVL	Treatment	Sample Collection Time after PVL	No. of Animals
1	Control	no	no	–	8
2	36 h	yes	no	36 h	8
3	3 d	yes	no	3 days	8
4	7 d	yes	no	7 days	8
5	36 h + FA	yes	FA (2.5 mg/kg)	36 h	8
6	36 h + saline	yes	saline	36 h	8
7	7 d + FA	yes	FA (2.5 mg/kg)	7 days	8
8	7 d + saline	yes	saline	7 days	8

FA—folinic acid.

## Data Availability

The data presented in this study are available in the article.

## References

[1] Mohamed F., Kallioinen M., Braun M., Fenwick S., Shackcloth M., Davies R.J., Committee G. (2020). Management of colorectal cancer metastases to the liver, lung or peritoneum suitable for curative intent: Summary of NICE guidance. Br. J. Surg..

[2] Cugat E., Mir-Labrador J., Cortese S., Pareja E., Fuster J., Santoyo J., Robles Campos R., Parrilla Paricio P. (2018). Cirugía hepática. Cirugía Hepática.

[3] García Pérez R. (2005). Estudio Comparativo de Regeneración Hepática Inducida por Técnica ALPPS Versus Ligadura Portal en un Modelo Experimental en Rata. Ph.D. Thesis.

[4] Soares Kevin C., Jarnagin W.R., Doherty G.M. (2020). Liver & Portal Venous System. Current Diagnosis and Treatment Surgery.

[5] Bingham G., Shetye A., Suresh R., Mirnezami R. (2020). Impact of primary tumour location on colorectal liver metastases: A systematic review. World J. Clin. Oncol..

[6] Moris D., Ronnekleiv-Kelly S., Kostakis I.D., Tsilimigras D.I., Beal E.W., Papalampros A., Dimitroulis D., Felekouras E., Pawlik T.M. (2018). Operative Results and Oncologic Outcomes of Associating Liver Partition and Portal Vein Ligation for Staged Hepatectomy (ALPPS) Versus Two-Stage Hepatectomy (TSH) in Patients with Unresectable Colorectal Liver Metastases: A Systematic Review and Meta-Anal. World J. Surg..

[7] Lang H., Baumgart J., Mittler J. (2018). Associating Liver Partition and Portal Vein Ligation for Staged Hepatectomy in the Treatment of Colorectal Liver Metastases: Current Scenario. Dig. Surg..

[8] Hernandez-Alejandro R., Sharma H. (2016). Small-for-size syndrome in liver transplantation: New horizons to cover with a good launchpad. Liver Transplant..

[9] González Arribas M. (2019). Efecto de la Hepatectomía Parcial Sobre el Desarrollo de Metástasis Hepáticas: Revisión Bibliográfica y Estudio Experimental en un Modelo Murino.

[10] Zhang G.Q., Zhang Z.W., Lau W.Y., Chen X.P. (2014). Associating liver partition and portal vein ligation for staged hepatectomy (ALPPS): A new strategy to increase resectability in liver surgery. Int. J. Surg..

[11] Schnitzbauer A.A., Lang S.A., Goessmann H., Nadalin S., Baumgart J., Farkas S.A., Fichtner-Feigl S., Lorf T., Goralcyk A., Hörbelt R. (2012). Right Portal Vein Ligation Combined With In Situ Splitting Induces Rapid Left Lateral Liver Lobe Hypertrophy Enabling 2-Staged Extended Right Hepatic Resection in Small-for-Size Settings. Ann. Surg..

[12] Truant S., Scatton O., Dokmak S., Regimbeau J.-M., Lucidi V., Laurent A., Gauzolino R., Castro Benitez C., Pequignot A., Donckier V. (2015). Associating liver partition and portal vein ligation for staged hepatectomy (ALPPS): Impact of the inter-stages course on morbi-mortality and implications for management. Eur. J. Surg. Oncol..

[13] Khuu D.N., Nyabi O., Maerckx C., Sokal E., Najimi M. (2013). Adult Human Liver Mesenchymal Stem/Progenitor Cells Participate in Mouse Liver Regeneration after Hepatectomy. Cell Transplant..

[14] Wabitsch S., Benzing C., Krenzien F., Splith K., Haber P.K., Arnold A., Nösser M., Kamali C., Hermann F., Günther C. (2019). Human Stem Cells Promote Liver Regeneration After Partial Hepatectomy in BALB/C Nude Mice. J. Surg. Res..

[15] Li D.-L., He X.-H., Zhang S.-A., Fang J., Chen F.-S., Fan J.-J. (2013). Bone Marrow-Derived Mesenchymal Stem Cells Promote Hepatic Regeneration after Partial Hepatectomy in Rats. Pathobiology.

[16] Schadde E., Tsatsaris C., Swiderska-Syn M., Breitenstein S., Urner M., Schimmer R., Booy C., Z’graggen B.R., Wenger R.H., Spahn D.R. (2017). Hypoxia of the growing liver accelerates regeneration. Surgery.

[17] Maeno H., Ono T., Dhar D.K., Sato T., Yamanoi A., Nagasue N. (2005). Expression of hypoxia inducible factor-1α during liver regeneration induced by partial hepatectomy in rats. Liver Int..

[18] Schmeding M., Rademacher S., Boas-Knoop S., Roecken C., Lendeckel U., Neuhaus P., Neumann U.P. (2010). rHuEPo Reduces Ischemia-Reperfusion Injury and Improves Survival After Transplantation of Fatty Livers in Rats. Transplantation.

[19] Ohtake M., Aono T., Sakaguchi T. (1994). Liver regeneration is enhanced by omeprazole in rats following partial hepatectomy. Br. J. Surg..

[20] Portugal V., García-Alonso I., Méndez J. (1996). Hepatotrophic effect of folinic acid in rats. J. Surg. Res..

[21] Reddy S.K., Barbas A.S., Turley R.S., Steel J.L., Tsung A., Marsh J.W., Geller D.A., Clary B.M. (2011). A standard definition of major hepatectomy: Resection of four or more liver segments. HPB.

[22] Khan A.S., Garcia-Aroz S., Ansari M.A., Atiq S.M., Senter-Zapata M., Fowler K., Doyle M.B., Chapman W.C. (2018). Assessment and optimization of liver volume before major hepatic resection: Current guidelines and a narrative review. Int. J. Surg..

[23] Schroeder R.A., Marroquin C.E., Bute B.P., Khuri S., Henderson W.G., Kuo P.C. (2006). Predictive indices of morbidity and mortality after liver resection. Ann. Surg..

[24] Horiguchi N., Ishac E.J.N., Gao B. (2007). Liver regeneration is suppressed in alcoholic cirrhosis: Correlation with decreased STAT3 activation. Alcohol.

[25] Koteish A., Yang S., Lin H., Huang J., Diehl A.M. (2002). Ethanol Induces Redox-Sensitive Cell-Cycle Inhibitors and Inhibits Liver Regeneration After Partial Hepatectomy. Alcohol. Clin. Exp. Res..

[26] Cardin R., D’errico A., Fiorentino M., Cecchetto A., Naccarato R., Farinati F. (2002). Hepatocyte proliferation and apoptosis in relation to oxidative damage in alcohol-related liver disease. Alcohol Alcohol..

[27] De Santibañes M., Boccalatte L., de Santibañes E. (2017). A literature review of associating liver partition and portal vein ligation for staged hepatectomy (ALPPS): So far, so good. Updates Surg..

[28] Fischer C., Melstrom L.G., Arnaoutakis D., Jarnagin W., Brown K., D’Angelica M., Covey A., DeMatteo R., Allen P., Kingham T.P. (2013). Chemotherapy after portal vein embolization to protect against tumor growth during liver hypertrophy before hepatectomy. JAMA Surg..

[29] Zhao J., Xu H., Li Y., Gong L., Zheng G., Wang X., Luan W., Li S., Ma F., Ni L. (2019). NAFLD Induction Delays Postoperative Liver Regeneration of ALPPS in Rats. Dig. Dis. Sci..

[30] Almau Trenard H.M., Moulin L.E., Padin J.M., Gondolesi G.E., Barros Schelotto P. (2014). Desarrollo de un modelo experimental de ligadura portal asociada a transección parenquimatosa (ALPPS) en ratas. Cirugía Española.

[31] Yang X., Yang C., Qiu Y., Shen S., Kong J., Wang W. (2019). A preliminary study of associating liver partition and portal vein ligation for staged hepatectomy in a rat model of liver cirrhosis. Exp. Ther. Med..

[32] Shi J.H., Hammarström C., Grzyb K., Line P.D. (2017). Experimental evaluation of liver regeneration patterns and liver function following ALPPS. BJS Open.

[33] García-Pérez R., Revilla-Nuin B., Martínez C.M., Bernabé-García A., Baroja Mazo A., Parrilla Paricio P. (2015). Associated Liver Partition and Portal Vein Ligation (ALPPS) vs selective Portal Vein Ligation (PVL) for staged hepatectomy in a rat model. Similar regenerative response?. PLoS ONE.

[34] Liao M., Zhang T., Wang H., Liu Y., Lu M., Huang J., Zeng Y. (2017). Rabbit model provides new insights in liver regeneration after transection with portal vein ligation. J. Surg. Res..

[35] Croome K.P., Mao S.A., Glorioso J.M., Krishna M., Nyberg S.L., Nagorney D.M. (2015). Characterization of a porcine model for associating liver partition and portal vein ligation for a staged hepatectomy. HPB.

[36] Yifan T., Ming X., Yifan W., Hanning Y., Guangyi J., Peijian Y., Ke W., Xiujun C. (2019). Hepatic regeneration by associating liver partition and portal vein ligation for staged hepatectomy (ALPPS) is feasible but attenuated in rat liver with thioacetamide-induced fibrosis. Surgery.

[37] Dili A., Lebrun V., Bertrand C., Leclercq I.A. (2019). Associating liver partition and portal vein ligation for staged hepatectomy: Establishment of an animal model with insufficient liver remnant. Lab. Investig..

[38] Higgins G.M., Anderson R.M. (1931). Experimental pathology of the liver. Restoration of the liver of the white rat following partial surgical removal. AMA Arch. Pathol..

[39] Veith N.T., Histing T., Menger M.D., Pohlemann T., Tschernig T. (2017). Helping prometheus: Liver protection in acute hemorrhagic shock. Ann. Transl. Med..

[40] Correa-Gallego C., Tan K.S., Arslan-Carlon V., Gonen M., Denis S.C., Langdon-Embry L., Grant F., Kingham T.P., DeMatteo R.P., Allen P.J. (2015). Goal-Directed Fluid Therapy Using Stroke Volume Variation for Resuscitation after Low Central Venous Pressure-Assisted Liver Resection: A Randomized Clinical Trial. J. Am. Coll. Surg..

[41] Sheng R.F., Yang L., Jin K.P., Wang H.Q., Liu H., Ji Y., Fu C.X., Zeng M.S. (2018). Assessment of liver regeneration after associating liver partition and portal vein ligation for staged hepatectomy: A comparative study with portal vein ligation. HPB.

[42] Linecker M., Stavrou G.A., Oldhafer K.J., Jenner R.M., Seifert B., Lurje G., Bednarsch J., Neumann U., Capobianco I., Nadalin S. (2016). The ALPPS Risk Score: Avoiding Futile Use of ALPPS. Ann. Surg..

[43] Olthof P.B., Tomassini F., Huespe P.E., Truant S., Pruvot F.-R., Troisi R.I., Castro C., Schadde E., Axelsson R., Sparrelid E. (2017). Hepatobiliary scintigraphy to evaluate liver function in associating liver partition and portal vein ligation for staged hepatectomy: Liver volume overestimates liver function. Surgery.

[44] Kang D., Schadde E. (2017). Hypertrophy and Liver Function in ALPPS: Correlation with Morbidity and Mortality. Visc. Med..

[45] Wang M.J., Chen F., Lau J.T.Y., Hu Y.P. (2017). Hepatocyte polyploidization and its association with pathophysiological processes. Cell Death Dis..

[46] Fausto N., Campbell J.S. (2003). The role of hepatocytes and oval cells in liver regeneration and repopulation. Mech. Dev..

[47] Hwang S.Y., Kang Y.J., Sung B., Jang J.Y., Hwang N.L., Oh H.J., Ahn Y.R., Kim H.J., Shin J.H., Yoo M. (2018). Folic acid is necessary for proliferation and differentiation of C2C12 myoblasts. J. Cell. Physiol..

[48] Bayer A.L., Fraker C.A. (2017). The Folate Cycle As a Cause of Natural Killer Cell Dysfunction and Viral Etiology in Type 1 Diabetes. Front. Endocrinol. Lausanne.

[49] Williams P.J., Bulmer J.N., Innes B.A., Broughton Pipkin F. (2011). Possible Roles for Folic Acid in the Regulation of Trophoblast Invasion and Placental Development in Normal Early Human Pregnancy. Biol. Reprod..

[50] Portugal V. (1992). Estudio de la Regeneración Hepatocitaria en el Hígado Sometido a Isquemia Normotérmica. Ph.D. Thesis.

[51] Blomgren H., Edler D., Hallström M., Ragnhammar P. (1996). Dual effects of folinic acid in 5-fluorouracil induced killing of human tumor cell lines in vitro. Anticancer Res..

[52] Roncalés M., Achón M., Manzarbeitia F., Maestro de las Casas C., Ramírez C., Varela-Moreiras G., Pérez-Miguelsanz J. (2004). Folic Acid Supplementation for 4 Weeks Affects Liver Morphology in Aged Rats. J. Nutr..

[53] Marsillach J., Ferré N., Camps J., Riu F., Rull A., Joven J. (2008). Moderately High Folic Acid Supplementation Exacerbates Experimentally Induced Liver Fibrosis in Rats. Exp. Biol. Med..

